# Potent Anti-Platelet Constituents from *Centaurea iberica*

**DOI:** 10.3390/molecules16032053

**Published:** 2011-02-28

**Authors:** Amna Nisar Khan, Itrat Fatima, Urooj Abdul Khaliq, Abdul Malik, Ghulam Abbas Miana, Zia-ur-Rehman Qureshi, Huma Rasheed

**Affiliations:** 1 International Centre for Chemical and Biological Sciences, HEJ Research Institute of Chemistry, University of Karachi, Karachi 75270, Pakistan; 2 Riphah Institute of Pharmaceutical Sciences, Riphah International University, 7^th^ Avenue, G-7/4 Islamabad, Pakistan; 3 International Centre for Chemical and Biological Sciences, Dr. Panjwani Centre for Molecular Medicine and Drug Research, University of Karachi, Karachi 75270, Pakistan

**Keywords:** *Centaurea iberica*, nitrogenous compounds, dimeric lignan glucoside, anti platelet activity

## Abstract

New naturally occurring nitrogenous compounds **1** and **2**, along with a new dimeric lignan glucoside **3**, have been isolated from the ethyl acetate soluble fraction of *Centaurea iberica*. Their structures have been elucidated through spectroscopic techniques. All the isolated compounds showed significant platelet aggregation inhibition.

## 1. Introduction

The genus *Centaurea* belongs to the family Asteraceae and comprises several hundred species which are predominantly distributed around the Mediterranean area and Western Asia [1]. Many of these species have been used in traditional medicine for the treatment of cancer [2], microbial infections [3,4] and as stimulant, tonic [5,6], antadiabetic [7,8,9], diuretic [7] and antirheumatic [10,11]. *Centaurea iberica* is a herb commonly found in the northern areas of Pakistan. Previously, flavones, steroid, fatty acids, volatile constituents, sesquiterpene lactones and other terpenoids have been reported from this species [12,13,14,15,16]. Several interesting biological activities of this species have also been reported, including the anti-inflammatory, wound healing and insulin secretion activities of the plant extract [17,18]. The chemotaxanomic and ethnopharmacological importance of the genus *Centaurea* prompted us to undertake further phytochemical studies on *C. iberica*. An ethanolic extract of this plant showed strong toxicity in a brine shrimp lethality test [19]. Further fractionation revealed strong toxicity in the ethyl acetate and *n*-butanol soluble sub-fractions. Pharmacological screening of the ethyl acetate soluble sub-fraction revealed significant inhibition of platelet aggregation. A series of chromatographic resolutions applied on this fraction have now resulted in the isolation and structural elucidation of two new naturally occurring nitrogenous compounds, namely 3-methyl-2-benzyl-4-quinazolone (**1**) and methyl-2-[(methylamino) carbonyl] benzoate (**2**), along with a new dimeric lignan glucoside **3**, respectively. Compounds **1**–**3** showed significant inhibition of platelet aggregation.

## 2. Results and Discussion

The EtOAc soluble sub-fraction of the ethanolic extract of the whole plant of *Centaurea iberica* was subjected to a series of column chromatographic techniques to obtain compounds **1**–**3** and their structures established by UV, IR, MS and NMR spectroscopy.

Compound **1** was isolated as crystalline solid which gave a positive test for nitrogenous compounds with Dragendorf’s reagent. The molecular formula C_16_H_14_N_2_O was established by HREIMS showing M^+^ peak at *m/z* 250.1106 (calc. 250.1115). In the IR spectrum the bands at 1,678 cm^−1^ and 1585 cm^−1^ were characteristic of the quinazolone system [20], while another band at 1,412 cm^−1^ could be attributed to a N-Me functionality. The UV spectrum showed absorption maxima at 227, 269, 273 and 311 nm, also characteristic of a quinazolone system [21]. The EIMS gave a distinct molecular ion peak at *m/z* 250, while two other characteristic fragment ions at *m/z* 159 (M^+^–CH_2_C_6_H_5_) and 91 (CH_2_C_6_H_5_) revealed the presence of a benzylic moiety. The ^13^C-NMR (BB and DEPT) spectra of **1** corroborated the presence of one methyl, one methylene, nine methine and five quaternary carbons. The downfield signal at δ 164.0 could be attributed to the carbonyl carbon of the amide moiety while the signal at δ 28.2 could be assigned to the methyl group attached to nitrogen. The N-substituted aromatic carbon resonated at δ 150.0 while the amide substituted aromatic carbon appeared at δ 121.8. 

**Figure 1:**
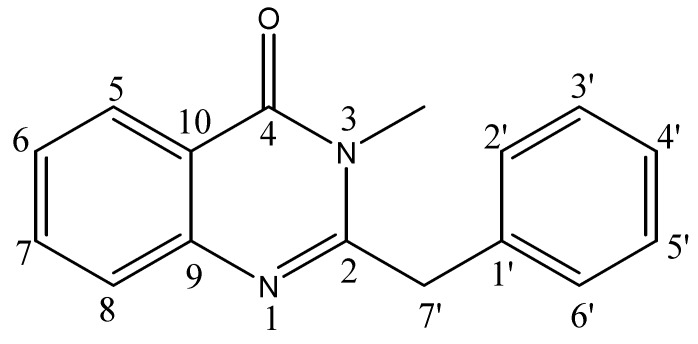
Structure of compound **1**.

The ^1^H-NMR spectrum showed the characteristic signals of the aromatic protons of the quinazolone system [δ 8.16 (1H, dd, *J* = 8.0, 1.5 Hz, H-5), 7.79 (1H, ddd, *J* = 8.0, 7.0, 1.5 Hz, H-7), 7.67 (1H, dd, *J* = 1.5, 8.0 Hz, H-8) and 7.48 (1H, ddd, *J* = 8.0, 7.0, 1.5 Hz, H-6)]. The assignments were corroborated with the help of ^1^H-^1^H COSY and HMBC correlations (Table 1). The two homotopic benzylic protons were observed as singlet at δ 4.00. The aromatic protons of the benzylic moiety were observed between δ 7.35–7.30. Its position was confirmed by HMBC showing ^2^*J* correlation of the benzylic protons at δ 4.00 with C-2 (δ 159.0). On the basis of these cumulative evidences, the structure of compound **1** could be assigned as 3-methyl-2-benzyl-4-quinazolone (Figure 1). This is the first report of the isolation of this compound from a natural source, following an earlier synthesis involving selective methylation of glycosminine [20].

**Figure 2:**
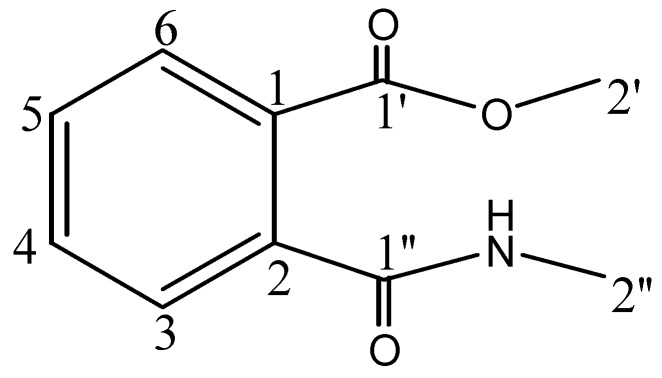
Structure of compound **2**.

Compound **2** was obtained as a white crystalline solid. Its HREIMS gave an M^+^ peak at *m/z* 193.0738 consistent with the molecular formula C_10_H_11_NO_3_ (calc. 193.0744). The IR spectrum revealed the presence of amide NH (3,400 cm^−1^), an ester (1,695 cm^−1^), amide carbonyl (1,675 cm^−1^), aromatic (1,500, 1,455 cm^−1^) and N- CH_3_ (1,414 cm^−1^) functionalities. The ^13^C-NMR spectra (BB and DEPT, Table 2) showed 10 signals, comprising two methyl, four methine and four quaternary carbons. The downfield signals at δ 171.4 and 169.7 were due to conjugated ester and amide moieties, respectively. A singlet at δ 52.9 was characteristic of a methoxyl carbon, while another singlet at δ 24.8 could be ascribed to N-CH_3_. The di-substituted aromatic ring could be confirmed by downfield signals at δ 141.7 and 131.5. The ^1^H-NMR spectrum (Table 2) showed a singlet at δ 4.88 for the amide proton while two further singlets at δ 3.91 and 2.19 were due to O-CH_3_ and N-CH_3_, respectively. The aromatic protons gave characteristic signals of 1, 2-di-substituted benzene ring with double doublets at δ 8.43 (*J* = 8.5, 1.5 Hz, H-3) 8.40 (*J* = 8.0, 1.5 Hz, H-6) and doublet of double doublets at δ 7.52 (*J* = 8.5, 7.0, 1.5 Hz, H-4) and 7.12 (*J* = 8.0, 7.0, 1.5 Hz, H-5). On the basis of these evidences along with HMQC and HMBC correlations, the structure of compound **2** could be assigned as methyl-2-[(methylamino)carbonyl]benzoate (Figure 2). This is the first report of natural occurrence of this compound following its earlier preparation via synthetic route [22].

Compound **3** was obtained as a yellow gum. Its molecular formula was established as C_54_H_66_O_21_ by HRFABMS, which showed an [M+H]^+^ peak at *m/z* 1051.4174 (calc. 1051.4179). The molecular formula was further confirmed through elemental analysis and low resolution ESI-MS showing [M+H]^+^ peak at *m/z* 1,051. The IR spectrum showed hydroxyl group (3,400 cm^−1^), a γ-lactone (1,760 cm^−1^) and aromatic (1,590 and 1,514 cm^−1^) functionalities. The UV absorption bands at 228 and 280 nm were assignable to the multiple substituted aromatic rings. The ^1^H-NMR spectrum (Table 3) was consistent with those of *trans*-dibenzylbutyro lactone derivatives [23]. The benzylic protons at δ C-7 with H-8 and H-8′ give a complex multiplet at δ 2.48–2.67, while the other benzylic protons (H_2_-7′) are more de-shielded due to the proximity of the lactone carbonyl and appear as double doublets at δ 2.98 and 2.80. The two protons at C-9 resonated characteristically as double doublets, at δ 4.18 and 3.93. The rest of the spectrum revealed the presence of six aromatic protons and three methoxyl groups. These were consistent with the presence of a guaiacyl ring and a vertaryl ring in the molecule. It was also supported by EIMS which showed prominent fragments at *m/z* 372, 151 and 137 corresponding to benzyl fragments, one of these being substituted with one hydroxy and one methoxy groups and the other containing the remaining methoxy groups.

**Figure 3:**
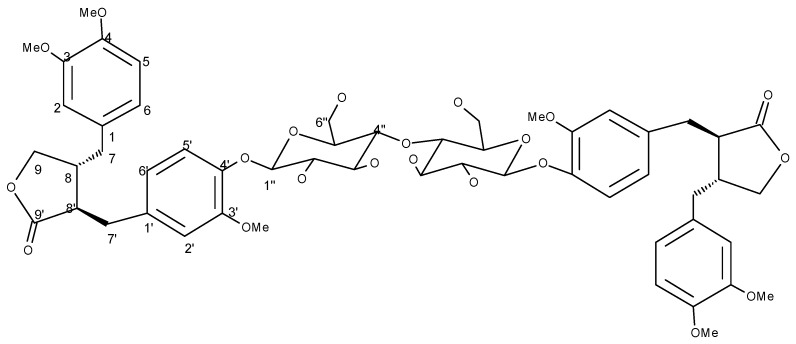
Structure of compound **3**.

The presence of a hexose moiety was evident by an anomeric proton at δ 4.88 (*J* = 7.5 Hz). Its larger coupling constant was in conformity to the *β*-linkage of the sugar moiety. Other oxymethine and oxymethylene protons are shown in Table 3. Acid hydrolysis of **3** provided d-glucose, which could be identified through the sign of its optical rotation and comparison of retention time of its TMS ether with a standard in GC. The HMBC correlation of anomeric proton at δ 4.88 with C-4 (δ 149.2) confirmed the point of attachment of the glucose moiety. This could be authenticated by close similarity of ^1^H- and ^13^C-NMR data of **3** with those of arctiin [24]. Insofar however, as the two compounds differ in specific rotations and the molecule has twice as many carbon atoms as arctin, compound **3** must be a symmetrical dimer involving two molecules of arctin. Concluding evidence was provided by acetylation of **3**, which provided a tetraacetyl derivative showing a [M+H]^+^ peak in HRFABMS at *m/z* 1,219.4597 (calc. 1,219.4604). The careful comparison of its ^1^H-NMR spectrum with that of **3** revealed downfield shifts of H-2″ and H-3″ due to the acetylation of both the hydroxyl groups at C-2″ and C-3″, respectively. On the other hand, H-4″ remained unaffected providing evidence that the hydroxyl groups at C-4″ of the sugar moieties of both the monomers are involved in ethereal band formation. Conclusive evidence was provided by dissolving compound **3** in D_2_O-CD_3_OD and repeating the ESI-MS which now gave an [M+H]^+^ peak at *m/z* 1057 due to deuterium exchange of six acidic alcoholic sugar protons [25]. Hence the structure of compound **3** could be assigned as illustrated in Figure 3. 

**Figure 4:**
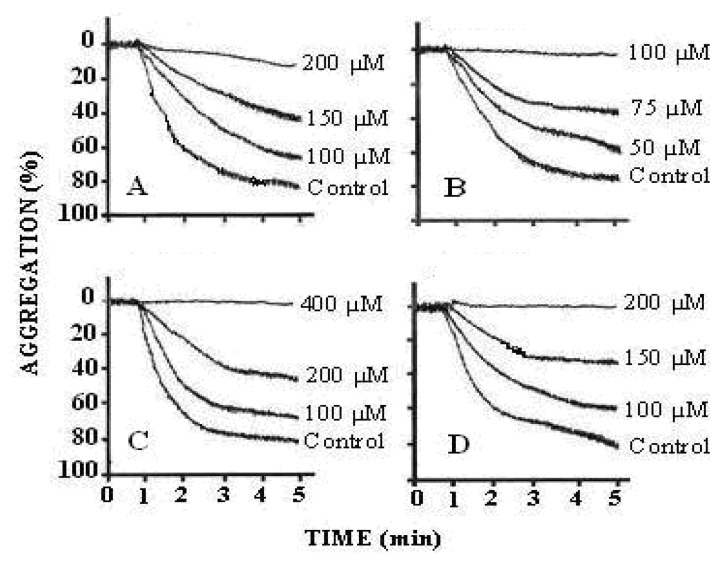
Tracings from the representative experiments showing the effect of various inhibitors on arachidonic acid induced platelet aggregation. (A = compound **1**; B = compound **2**; C = compound **3** and D = aspirin showing inhibition of arachidonic acid induced platelet aggregation in a dose-dependent manner. Control = arachidonic acid (1.73 mM), N = 8–10.

**Figure 5:**
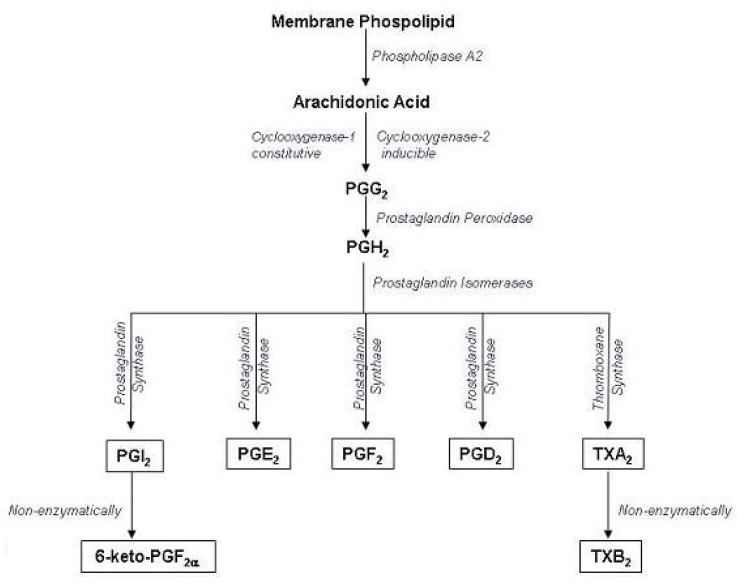
Schematic representation of arachidonic acid metabolism in platelets through cyclooxygenase that catalyzes the synthesis of prostaglandins, thromboxane A_2_ and prostacyclins.

The results of the antiplatelet activity demonstrated that treatment of PRP with arachidonic acid (0.5–1.7 mM) showed a concentration-dependent increase in platelet aggregation. The maximum aggregation was obtained with 1.73 mM of arachidonic acid. We found that the effect of arachidonic acid was inhibited by pre-treatment of PRP with compounds **1-3**. All of these compounds inhibited human platelet aggregation in a concentration dependent manner (compound **1** = 100, 150 and 200 μM; compound **2** = 50, 75 and 100 μM and compound **3** = 100, 200 and 400 μM) (Figure 4). The IC_50_ values are illustrated in Table 4. The results indicated that all the three compounds were active against arachidonic acid induced platelet aggregation as compared to the effect of aspirin, which inhibits TXA_2_ formation. TXA_2_ is a potent mediator of platelet aggregation released from platelets when incubated with arachidonic acid. The underlying mechanism by which the active metabolites of arachidonic acid (AA), *i.e*., thromboxane A_2_ and/or prostaglandin H_2_ (TXA_2_/PGH_2_) induce platelet aggregation is still unclear. Several reports have suggested that AA-stimulated aggregation is mediated by secreted ADP, whereas other studies have proposed that this response is ADP-independent. Arachidonic acid is the precursor of prostanoids which liberates from lipid membrane of platelets by the action of phospholipase A_2_. AA is processed in two stages by cyclooxygenase to produce prostaglandin (PG) H_2_. This process is aspirin-sensitive, as the drug irreversibly acetylates and inhibits cyclooxygenase that shows the basis for the effective use of aspirin as antithrombotic agent, which itself underscores its involvement in arterial thrombosis. Thromboxane synthase then consumes a proportion of PGH_2_ to generate thromboxane (TXA_2_) that activates platelet aggregation [26] (Figure 5).

It is proposed that these compounds may produce anti-platelet effects due to the inhibition of cycloxygenase enzyme responsible for the synthesis of thromboxane A_2_ like aspirin. Arachidonic acid was used as potent platelet aggregating agent which is the substrate of cycloxygenase enzyme that synthesizes thromboxane A_2_ in the platelets. It is the most potent mediator of platelet aggregation that releases from platelets and acts on its receptor on the surface of the platelets in an autocrine fashion and mediates platelet aggregation [27]. As we compared the effects of these compounds with aspirin that has the same mechanism in inhibiting COX enzyme followed by the inhibition of TXA_2_, we preferred arachidonic acid in this study as it has a major role in platelet. From the present in *vitro* studies it can be concluded that compounds **1-3** have potential for future development as anti platelet drugs. However, in *vivo* studies could not be carried out at this time due to paucity of materials.

## 3. Experimental

### 3.1. General

Optical rotations were measured on a JASCO DIP-360 polarimeter. IR spectra were recorded on a 460 Shimadzu spectrometer. The UV spectra were recorded on a Hitachi UV-3200 spectrometer. The HREIMS were recorded on JEOL JMX-HX 110 mass spectrometer while HRFABMS was recorded on JEOL JMS-DA-5000 mass spectrometer. ESI mass spectrum was obtained on QSTAR XL spectrometer. The ^1^H- and ^13^C-NMR, HMQC and HMBC spectra were recorded on Bruker spectrometer operating at 500 MHz for ^1^H– and 125 MHz for ^13^C-NMR, respectively. The chemical shift values are reported ppm (δ) units and *J* are in Hz. The parameters for 1D and 2D NMR spectra were similar those reported by us previously [28]. The elemental analysis was performed on a Perkin-Elmer elemental analyzer, model 240. Aluminum sheets pre-coated with silica gel 60 F_254_ (20 × 20 cm, 0.2 mm thick, E-Merck, Darmstadt, Germany) were used for thin layer chromatography (TLC) and silica gel (230–400 mesh, E-Merck, Darmstadt, Germany) was used for column chromatography. The purity of the compounds was routinely checked by TLC using HPTLC precoated glass plates of silica gel 60 F_254_ (20 × 10 cm., E. Merck, Darmstadt, Germany). Platelet aggregation was monitored on dual channel Lumi aggregometer (Model 400, Chronolog Corporation, Chicago, IL, USA). EtOH, AcOEt, *n*-hexane, *n*-butanol, Arachidonic acid and sodium citrate were purchased from Sigma Chemical Co. (St. Louis, MO, USA). All other chemicals used were of the highest purity grade. Arachidonic acid was reconstituted in ethanol and 0.2% w/v aqueous solution of sodium carbonate. The contents were then vortexed and transferred under nitrogen to screw capped vial to avoid auto-oxidation.

### 3.2. Plant Material

The whole plant material of *Centaurea iberica* Trev. was collected from Islamabad in April, 2007 and identified by Prof. S. Iftikhar Hussain Shah, Department of Pharmacognosy, Riphah International University, Islamabad. A voucher specimen has been deposited in the herbarium of Plant Sciences, Quaid-i-Azam University, Islamabad (voucher specimen no. ISL 49920).

### 3.3. Extraction and Isolation

The shade dried whole plant material (20 kg) was extracted with EtOH (3 × 50 L) at room temperature. The combined ethanolic extract was evaporated under reduced pressure to obtain a thick gummy residue (300 g) which was suspended in water and successively extracted with *n*-hexane, ethyl acetate and *n*-butanol. The ethyl acetate soluble fraction (100 g) was subjected to column chromatography eluting with mixtures of *n*-hexane, EtOAc and MeOH in increasing order of polarity to obtain six fractions (A-F). The fraction B obtained from *n*-hexane-EtOAc (8:2) was rechromatographed and eluted with *n*-hexane-EtOAc (7:3) to obtain compounds **1** (10 mg) and **2** (14 mg) from the head and tail fractions, respectively. The fraction F obtained from EtOAc-MeOH (9:1) was rechromatographed and eluted with mixtures of *n*-hexane, EtOAc and MeOH in increasing order of polarity. The fraction which eluted with EtOAc-MeOH (9:1) was a semi pure compound which was purified by further chromatography eluting with EtOAc-MeOH (9.2:0.8) to furnish compound **3** (20 mg).

*3-Methyl-2-benzyl-4-quinazolone* (**1**): Crystalline solid. UV (MeOH) λ_max_ (log *ε*_max_): 227 (3.25), 269 (3.01), 273 (3.42), 311 (2.78) nm. IR (KBr): γ = 1,678, 1,585, 1,412 cm^−1^. EIMS *m/z* (rel. int.): 250 (20), 236 (60.1), 159 (25), 91 (100). HREIMS: *m/z* 250.1106 (calcd. 250.1115 for C_16_H_14_N_2_O). ^1^H- and ^13^C-NMR: see Table 1.

*Methyl-2-[(methylamino)carbonyl]benzoate* (**2**): White crystalline solid. UV (MeOH) λ_max_ (log *ε*_max_): 208 (3.12), 231 (3.13), 169 (3.28) nm. IR (KBr): γ = 3,400, 1,695, 1,675, 1,500, 1,455, 1,414 cm^−1^. EIMS *m/z* (rel. int.): 193 (20), 179 (45), 120 (100). HREIMS: *m/z* 193.0738 (calcd. 193.0744 for C_10_H_11_NO_3_). ^1^H- and ^13^C-NMR: see Table 2.

*(3R,4R)-4-(3,4-dimethoxybenzyl)-3-(4-{[5-{[6-(4-{[(3R,4R)-4-(3,4-dimethoxybenzyl)-2-oxotetrahydro-3-furanyl]methyl}-2-methoxyphenoxy)-4,5-dihydroxy-2-(hydroxylmethyl)tetrahydro-2H-pyran-3-yl] oxy}-3,4-dihydroxy-6-(hydroxymethyl)tetrahydro-2H-pyran-2-yl]oxy}-3-methoxybenzyl)dihydro-2-(3H)-furanone* (**3**): Yellow gum. UV (MeOH) λ_max_ (log *ε*_max_): 228 (4.12), 280 (4.08) nm. IR (KBr): γ = 3,400, 1,760, 1,590, 1,514 cm^−1^. EIMS *m/z* (rel. int.): 372 (35), 151 (69), 137 (100). HRFABMS: m/z 1051.4174 (calcd. 1051.4179 for C_54_H_67_O_21_). ESIMS: *m/z* 1051 and *m/z* 1057 (after dissolving compound **3** in D_2_O-CD_3_OD). ^1^H- and ^13^C-NMR: see Table 3. Anal. Calcd. for C_54_H_66_O_21_: C, 61.71, H, 6.28, O,32.00; Found: C, 61.67, H, 6.23, O, 31. 89.

### 3.4. Acid Hydrolysis of Compound ***3***

A solution of compound **3** (4.2 mg) in MeOH (5 mL) containing 1N HCl (2 mL) was refluxed for 4 h, concentrated under reduced pressure, diluted with H_2_O and extracted with EtOAc. The organic phase provided the aglycone as colorless gummy solid which could be identified as arctigenin through comparison of spectral data with those reported in literature [24]. The aqueous phase was concentrated to obtain the glycone which could be identified as d-glucose by the sign of its optical rotation ([α]_D_^23^ + 51.8 (*c* = 0.02, MeOH) and comparison of retention time of its TMS ether with that of standard in gas chromatography. The preparation of TMS ether and conditions of GC were same as reported previously in literature [24].

### 3.5. Preparation of Human Platelets

Blood was drawn by vein-puncture from healthy human subjects who had not taken any medicine for at least one week. Each sample was mixed with 3.8% (w/v) sodium citrate solution (9:1) and centrifuged at 260 g for 15 min at 20 °C to obtain platelet rich plasma (PRP). Platelet count was determined by phase contrast microscopy and all aggregation studies were carried out at 37 °C with PRP having platelet count between 2.5 and 3.0 × 10^8^ mL^−1^ of plasma [27]. Platelet poor plasma (PPP) was obtained by centrifuging blood at 400 g for 5 min and was used as a blank source in the aggregometer. All experiments were performed within 2 h of PRP preparation. 

### 3.6. Measurement of Platelet Aggregation

Aggregation was monitored using Dual-channel Lumi-aggregometer using 0.45 mL aliquots of PRP in a glass cuvette. The final volume was made up to 0.5 mL with the test drug dissolved either in normal saline or appropriate vehicle known to be devoid of any effect on aggregation. Dual-channel Lumi-aggregometer works on the principle of light transmission. The more platelet are activated the greater aggregation is observed after adding the stimulating agent. The test compounds were dissolved in DMSO in such a way that the final concentration in cuvette was less than 1% as DMSO itself has an inhibitory effect on platelets. Aggregation was induced with arachidonic acid (1.73 mM) and the anti platelet activity of different compounds were studied by pretreatment of PRP with various concentrations of the inhibitors for 1 min followed by the addition of arachidonic acid. The resulting aggregation was recorded for 5 min after challenge by the change in light transmission as a function of time. Once the anti platelet activity of various inhibitors against agonists was established, dose response curves were constructed to calculate the IC_50_ values of the agonists and compounds. Aspirin, a potent anti-platelet agent was taken as a reference drug. 

### 3.7. Data Analysis

IC_50_ = Concentration (*μ*M) producing 50% inhibition of platelet aggregation (control response taken as 100%). The IC_50_ were calculated as means ± SEM of 5–6 independent experiments. Differences between control and test measurements were assessed by student’s t-test. 

## 4. Conclusions

In conclusion, the new naturally occurring nitrogenous compounds **1** and **2**, along with a new dimeric lignan glucoside **3**, have been isolated from *C. iberica* and their structures have been elucidated through spectroscopic techniques. All the isolated compounds showed significant inhibition of platelet aggregation. 

## Figures and Tables

**Table 1:** ^1^H- (500 MHz) and ^13^C-NMR (125 MHz) data of **1** (CD_3_OD, *J* in Hz and δ in ppm) and HMBC correlations.

Position	^1^H	^13^C	HMBC (correlation with ^1^H)
1	-	-	-
2	-	159.0	-
3	-	-	-
4	-	164.5	-
5	8.16 (dd, 8.0, 1.5)	127.1	4, 6, 7, 9, 10
6	7.48 (ddd, 8.0, 7.0, 1.5)	127.9	5, 7, 8
7	7.79 (ddd, 8.0, 7.0, 1.5)	136.0	5, 6, 8, 9
8	7.76 (dd, 8.0, 1.5)	127.5	6, 7, 9, 10
9	-	150.0	-
10	-	121.8	-
1′	-	137.1	-
2′/6′	7.30 (m)	129.9	1′,3′,4′,5′
3′/5′	7.35 (m)	129.8	1′, 2′, 4′, 6′
4′	7.23 (m)	128.3	2′,3′,5′,6′
7′	4.00 (s)	42.3	2,1′,2′,6′
N-Me	1.88 (s)	28.2	2, 4

**Table 2:** ^1^H- (500 MHz) and ^13^C-NMR (125 MHz) data of **2** (CD_3_OD, *J* in Hz and δ in ppm) and HMBC correlations.

Position	^1^H	^13^C	HMBC (correlation with ^1^H)
1	-	141.7	-
2	-	131.3	-
3	8.43 (dd, 8.5, 1.5)	122.0	1, 2, 4, 5, 1′
4	7.52 (ddd, 8.5, 7.0, 1.5)	135.2	2, 3, 5, 6
5	7.12 (ddd, 8.0, 7.0, 1.5)	124.2	1, 3, 4, 6
6	8.43 (dd, 8.0, 1.5)	131.9	1, 2, 4, 5
1′	-	171.4	-
2′	3.91 (s)	52.9	1′
1″	-	169.7	-
2″	2.19 (s)	24.8	1″

**Table 3:** ^1^H- (500 MHz) and ^13^C-NMR (125 MHz) data of **3** (CD_3_OD, *J* in Hz and δ in ppm) and HMBC correlations.

Positions	^1^H	^13^C	HMBC (correlation with ^1^H)
1	-	132.7	-
2	6.74 (d, 1.5)	114.8	1, 3, 4, 6, 7
3	-	150.7	-
4	-	149.2	-
5	6.81 (d, 8.0)	113.1	1, 3, 4, 6
6	6.59 (br s)	122.1	1, 2, 4, 5, 7
7	2.53 (m)	38.9	8′, 1, 2, 6, 8, 9
8	2.48 (m)	42.5	7′, 8′, 9′, 1, 7, 9
9	4.18 (dd, 7.5, 9.0) 3.93 (dd, 7.5, 9.0)	72.9	8′, 9′, 7, 8
1′	-	134.3	-
2′	6.61 (d, 1.5)	113.6	1′, 3′, 4′, 6′, 7′
3′	-	150.4	-
4′	-	146.9	-
5′	7.03 (d, 8.5)	117.8	1′, 3′, 4′, 6′
6′	6.63 (dd, 8.0, 1.5)	123.0	1′, 2′, 4′, 5′, 7′
7′	2.98 (dd, 5.0, 13.5) 2.80 (dd, 7.5, 13.5)	35.4	1′, 2′, 6′, 8′, 9′, 8
8′	2.67 (m)	47.6	1′, 7′, 9′, 7, 8, 9
9′	-	181.3	-
1″	4.88 (d, 7.5)	102.9	4′, 2″, 3″
2″	3.47 (m)	74.9	1″, 3″, 4″
3″	3.93 (m)	78.1	1″, 2″, 4″, 5″
4″	3.38 (m)	71.3	2″, 3″, 5″, 6″
5″	3.46 (m)	77.8	3″, 4″, 6″
6″	3.66 (br d, 12.5) 3.84 (br d, 12.5)	62.5	4″, 5″
3-OMe	3.79 (s)	56.5	3
4-OMe	3.79 (s)	56.5	4
3′-OMe	3.74 (s)	56.7	3′

**Table 4:** The effects of compounds **1-3** on arachidonic acid (1.73 mM) induced platelet aggregation.

Inhibitors	Mean IC_50_ *μ*M ± SEM
Compound **1** Compound **2** Compound **3** Aspirin	160 ± 3.5 75 ± 2.1 45 ± 2.8 150 ± 4.4

Data is mean ± SEM (n = 8–10) and is indicated as half-maximal effect (IC_50_) of the inhibitors.
